# Adjuvant Radiotherapy Significantly Increases Neck Control and Survival in Early Oral Cancer Patients with Solitary Nodal Involvement: A National Cancer Registry Database Analysis

**DOI:** 10.3390/cancers13153742

**Published:** 2021-07-26

**Authors:** Chia-Jen Tsai, Yu-Hsuan Kuo, Hung-Chang Wu, Chung-Han Ho, Yi-Chen Chen, Ching-Chieh Yang

**Affiliations:** 1 Department of Radiation Oncology, Chi Mei Medical Center, Tainan 71069, Taiwan; b101100015@tmu.edu.tw; 2 Division of Hematology and Oncology, Department of Internal Medicine, Chi Mei Medical Center, Tainan 71069, Taiwan; beethovan@gmail.com (Y.-H.K.); 980805@mail.chimei.org.tw (H.-C.W.); 3 Department of Cosmetic Science, Chia-Nan University of Pharmacy and Science, Tainan 71710, Taiwan; 4 Department of Pharmacy, Chia-Nan University of Pharmacy and Science, Tainan 71710, Taiwan; 5 Department of Medical Research, Chi Mei Medical Center, Tainan 71069, Taiwan; a01111@mail.chimei.org.tw (C.-H.H.); laura751111986@hotmail.com (Y.-C.C.); 6 Department of Information Management, Southern Taiwan University of Science and Technology, Tainan 710301, Taiwan

**Keywords:** oral cancer, pN1, adjuvant radiotherapy, neck control, survival

## Abstract

**Simple Summary:**

For early oral cancer with solitary nodal metastasis, the role of adjuvant radiotherapy on neck control and survival remains controversial. Based on our national cancer registry database, univariate and multivariable analysis showed that adjuvant radiotherapy significantly associated with better 5-year OS and DFS compared to patients who received surgery alone. Stratified analysis indicated the greatest survival advantage for 5-year OS and DFS in those with pT2 classification and non-tongue disease. Moreover, adjuvant radiotherapy significantly protected against neck recurrence.

**Abstract:**

We assessed the role of adjuvant radiotherapy on neck control and survival in patients with early oral cancer with solitary nodal involvement. We identified pT1-2N1 oral cancer patients with or without adjuvant radiotherapy from the 2007–2015 Taiwan Cancer Registry database. The effect of adjuvant radiotherapy on 5-year neck control, overall survival (OS) and disease-free survival (DFS) were calculated using the Kaplan–Meier method, log-rank tests, and Cox regression analysis. Of 701 patients identified, 505 (72.0%) received adjuvant radiotherapy and 196 (28.0%) had surgery alone. Patients receiving adjuvant radiotherapy were more likely to be aged <65 years, pT2 stage, poorly graded and without comorbid conditions (all, *p* < 0.05). The 5-year OS and DFS differed significantly by receipt of adjuvant radiotherapy. Multivariable analysis showed adjuvant radiotherapy significantly associated with better 5-year OS (adjusted hazard ratio (aHR), 0.72; 95% confidence interval (CI), 0.54–0.97; *p* = 0.0288) and DFS (aHR, 0.64; 95% CI, 0.48–0.84; *p* = 0.0016). Stratified analysis indicated the greatest survival advantage for both 5-year OS and DFS in those with pT2 classification (*p* = 0.0097; 0.0009), and non-tongue disease (*p* = 0.0195; 0.0158). Moreover, adjuvant radiotherapy significantly protected against neck recurrence (aHR, 0.30; 95% CI, 0.18–0.51; *p* < 0.0001). Thus, adjuvant radiotherapy is associated with improved neck control and survival in these early oral cancer patients.

## 1. Introduction

Oral cancer remains one of the most common cancers worldwide [1]. In Taiwan, with a high prevalence rate of the habits of smoking and betel nut chewing, oral cancer is the fourth highest malignancy among men and ranks fifth among the top 10 causes of cancer-related mortality [2]. Although approximately 50% of oral cancer patients are diagnosed with an early stage of the disease and achieve excellent results, many studies have reported locoregional recurrence rates of 30–35% [3]. Moreover, when positive nodal disease is initially confirmed, the 5-year survival rate decreases by 10–20% compared to those without neck metastasis [4]. Thus, appropriate initial management for early oral cancer is crucial.

For oral cancer management, adjuvant radiotherapy is often used to prevent locoregional failure and improve survival [5]. According to current National Comprehensive Cancer Network guidelines for head and neck cancers, adjuvant radiotherapy is recommended to patients with advanced-stage disease (pT3-4 or N2-3), close/positive margins, perineural or vascular invasion, or extra-nodal extension [6]. However, the benefit of adjuvant radiotherapy for patients with early oral cancer with solitary nodal involvement (pT1-2N1) remains controversial [7,8,9]. The main reasons for this gap in knowledge may include the small sample sizes of previous studies, their use of old databases, or their failure to include important information such as radiation fields and the quality of neck dissection. Therefore, in this study, we aimed to assess whether adjuvant radiotherapy improves neck control and survival in patients with pT1-2N1 oral cancer by utilizing the national Taiwan Cancer Registry (TCR) database, which includes comprehensive information, to help us investigate this important issue.

## 2. Materials and Methods

### 2.1. Data Source

Based on our national TCR and National Health Insurance (NHI) Research Database, early oral cancer patients with single nodal metastasis receiving radical surgery including primary tumor resection and neck dissection were identified from 2007 to 2015. Comparing to other well-established cancer registries, our TCR database includes 97% of the cancer cases in Taiwan and has excellent accuracy of diagnosis and treatment coding [10,11].

### 2.2. Patient Demographics and Measurements

The International Classification of Disease for Oncology, third edition code (ICD-O-3) was used to identify the diagnosis of oral cancer. The subsites included lip (excluding skin of lip) (C00), tongue (C02), gum (C03), floor of mouth (C04), palate (C05), and other parts of the mouth such as the buccal area (C06); histologic type: squamous cell carcinoma (M8050-M8084). Clinicopathological data included the date of diagnosis, site of the primary oral tumor, age, gender, margin status, histology grade, presence of extra-nodal extension, clinical/pathological tumor–node–metastasis (TNM) stage, neck lymph node (LN) yield, chemotherapy, radiotherapy, cause of death, comorbidities and hospital level. The cancer staging of all patients were modified to the American Joint Committee on Cancer classification system (7th edition). The Charlson Comorbidity Index (CCI), based on the International Classification of Diseases, 9th revision code, was used for severity grading [12]. In this study, we only included oral cancer patients with pT1-2N1 disease. This means that the tumors were not more than 4 cm and the nodal statuses were limited to a single node, ipsilateral and no larger than 3 cm. Patients with incomplete data, such as for age, gender, radiotherapy, stage, follow-up data, history of neoadjuvant therapy, adjuvant chemotherapy and those with inadequate neck dissection (LN yield < 18) were excluded [13]. We also excluded patients with margin-positive or extra-nodal extension disease due to these patients being indicated for chemoradiation instead of adjuvant radiotherapy alone according to the recommendations of current guidelines [5]. Finally, a total of 701 pT1-2N1 oral cancer patients with or without adjuvant radiotherapy were included in this analysis. In this study, the primary end points were the overall survival (OS) and disease-free survival (DFS) rates. Deaths due to cancer recorded as events and deaths secondary to other causes, at 5 years following diagnosis or the last follow-up date, were recorded as censored. By accessing existing data, 5-year neck recurrence was also analyzed.

### 2.3. Statistical Analysis

Pearson’s chi-square test for categorical variables and the Wilcoxon ranked sum test for continuous variables were performed for the distribution difference between oral cancer patients receiving adjuvant radiotherapy or not. The Kaplan–Meier method was applied to estimate 5-year OS and DFS rates. Log-rank tests were also used to compare the difference of survival curves. Cox proportional hazard models were fitted to estimate the effect of adjuvant radiotherapy on survival, after adjusting to other confounding variables. All statistical analyses were performed using SAS 9.4 for Windows (SAS Institute, Inc., Cary, NC, USA). The *p* value less than 0.05 was considered statistically significant.

## 3. Results

### 3.1. Clinicopathological Characteristics

The clinicopathological characteristics of the patients in this study are displayed in Table 1. A total of 701 patients were identified: 628 males (89.6%) and 73 females (10.4%). The mean age at diagnosis was 51.96 ± 10.82 years and the mean follow-up time was 3.53 ± 1.61 years. Among these patients, 505 (72.0%) received adjuvant radiotherapy and 196 (28.0%) had surgery alone. Patients who received adjuvant radiotherapy were more likely to be aged <65 years, be at pT2 stage, have poorly graded disease and have no comorbid conditions (CCI = 0) (all, *p* < 0.05); however, the two groups had no significant differences in gender, tumor sites or hospital level. In Table S1, the most common tumor subsite was oral tongue (*n* = 335, 47.79%), followed by other parts of the mouth (*n* = 302, 43.08%), floor of mouth (*n* = 28, 3.99%), gum (*n* = 20, 2.85%), and hard palate (*n* = 16, 2.28%). Radiation treatment parameters including RT dose, techniques and fields are summarized in Table 2. The median total dose of adjuvant radiotherapy was 63 Gy (IQR 60–66) in 33 fractions (IQR 30–34). Most patients received adjuvant radiotherapy with modern techniques (85.43%) such as IMRT (intensity modulated radiation therapy) or VMAT (volumetric modulated arc therapy) and the radiation field was both to the primary tumor and neck area (98.80%).

### 3.2. Analysis for Survival and Neck Control

**Figure 1:**
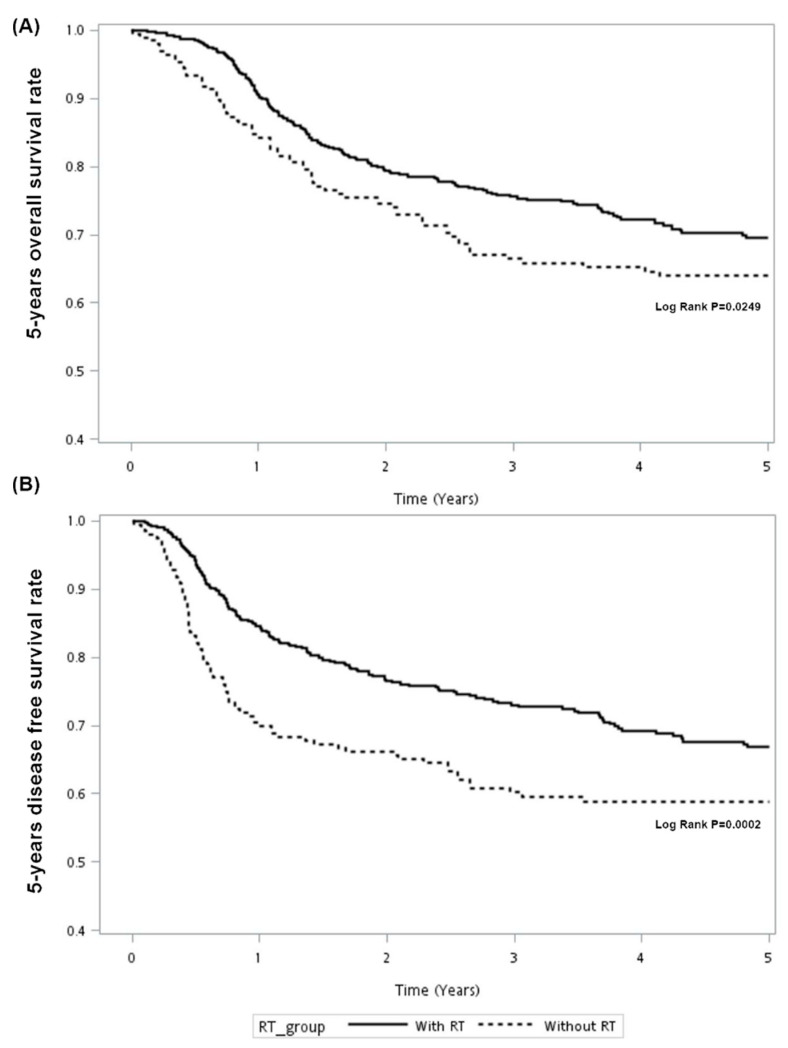
5-year overall survival rate (**A**) and disease-free survival (**B**) in oral cancer patients with or without adjuvant radiotherapy (RT).

**Figure 2:**
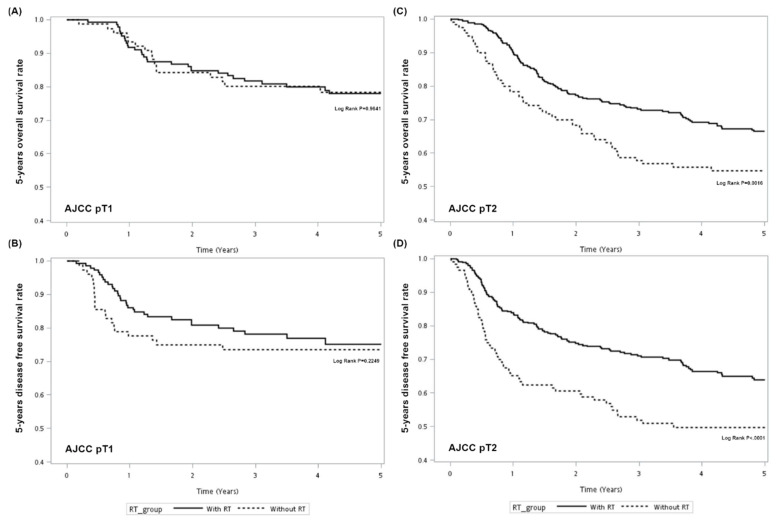
5-year overall survival rate (**A**,**C**) and disease-free survival (**B**,**D**) regarding different AJCC stages in oral cancer patients with or without adjuvant radiotherapy (RT).

Kaplan–Meier survival curves were generated to compare the 5-year OS and DFS by receipt of radiotherapy. As presented in Figure 1, the 5-year OS and DFS differed significantly between those who received adjuvant radiotherapy and those who received surgery alone. Log-rank tests confirmed that adjuvant radiotherapy improved the 5-year OS and DFS compared to surgery alone (*p* = 0.0249; *p* = 0.0002). However, after adjustment for confounders (Table 3), multivariable analysis indicated that adjuvant radiotherapy was significantly associated with better 5-year OS (adjusted hazard ratio (aHR), 0.72; 95% CI, 0.54–0.97; *p* = 0.0288) and DFS (aHR, 0.64; 95% CI, 0.48–0.84; *p* = 0.0016). In Figure 2, stratified analysis for 5-year OS and DFS according to different AJCC pT classifications indicated that the survival advantage for 5-year OS and DFS was the largest in those with pT2 classification (*p* = 0.016; < 0.0001), but not statistically significant for those with pT1 classification (*p* = 0.9641; 0.2249). The multivariate analysis shown in Table 4 indicated that adjuvant radiotherapy was a significant protective factor for 5-year OS in females (aHR, 0.31; 95% CI, 0.12-0.84; *p* = 0.0206), those aged ≥65 years (aHR, 0.29; 95% CI, 0.14–0.62; *p* = 0.0012), those with pT2 classification (aHR, 0.65; 95% CI, 0.46–0.90; *p* = 0.0097) and those with non-tongue disease (aHR, 0.62; 95% CI, 0.41–0.93; *p* = 0.0195). For 5-year DFS, the protective role of adjuvant radiotherapy was found in pT2 classification (aHR, 0.58; 95% CI, 0.42–0.80; *p* = 0.0009) and non-tongue disease (aHR, 0.61; 95% CI, 0.41–0.91; *p* = 0.0158). Unlike in previous studies, we aimed to differentiate the real effect of adjuvant radiotherapy on neck control and only included oral cancer patients with adequate neck dissection (LN ≥ 18). After excluding study patients with missing recurrent information (*N* = 35), we found that tumor relapse at the site of primary lesions occurred in 33 patients, with recurrences in the neck occurring in 43 cases, both local and regional recurrence in 17 patients and distant metastasis in 15 patients after initial surgery. Adjuvant radiotherapy played a significant protective role in neck recurrence (aHR, 0.30; 95% CI, 0.18–0.51; *p* < 0.001), as shown in Table 5.

## 4. Discussion

Nodal metastasis remains one of the most important prognostic indicators for outcomes in oral cancer, and adjuvant radiotherapy is often used for high-risk patients [14,15]. However, for early oral cancer with solitary nodal involvement, current evidence remains inconclusive [16,17,18]. This nationwide cancer registry database analysis indicated that adjuvant radiotherapy was significantly associated with better 5-year OS and DFS compared to patients who received surgery alone. Further stratified analysis revealed that adjuvant radiotherapy was a significant protective factor for 5-year OS in females, those aged ≥65 years, those with pT2 classification and those with non-tongue disease; for 5-year DSS, those with pT2 classification and non-tongue disease. Moreover, adjuvant radiotherapy could improve neck control even after adequate neck dissection.

Compared to previous research from single institutions or different national databases, our study has several strengths that make our results convincing. First, as we know, solitary nodal involvement is less common in patients with oral cancer than negative and multiple LN metastasis [9,14]. Based on this national database which includes over 95% of the cancer patients in Taiwan, the number of included patients was large (*N* = 701) and the follow-up duration was sufficient. In addition, we included patients with oral cancer diagnosed from 2007 to 2015, which made our study more reflective of current conditions. Second, this database had comprehensive information about radiotherapy (dose, fractions, techniques and field: primary, neck or both), allowing us to perform detailed analyses on the real effect of radiotherapy (Table 2). Third, inadequate neck dissection may underestimate the severity of nodal metastasis and incur insufficient cancer treatment [13]. Our study excluded all patients who had poor-quality neck dissection (harvested LN < 18), to ensure the accuracy of our outcomes. Finally, due to the information of comorbidities in our database, the acceptance to recommended radiotherapy and survival, especially to OS, could be assessed well.

Many studies investigated the role of adjuvant radiotherapy used to prevent locoregional recurrence and improve survival in pT1-2N1 oral cancer patients [7,8,9]. Jackel et al. reported data from a single institution of patients with head and neck cancer with pathological N1 neck disease. Among 55 patients with pT1-2 disease, those with or without adjuvant radiotherapy had a similar 5-year OS rate (63.6% vs. 60.7%, respectively, *p* = 0.6212) [8]. In a meta-analysis study, Moergel et al. found a marginally higher mortality rate after adjuvant radiotherapy (44% vs. 34%, respectively) in patients with pT1-2N1 oral cancer. However, their conclusion was vague due to a lack of valid and homogeneous outcome data [19]. Schiff et al. also reported that patients with pN1 oral tongue cancer who received adjuvant radiotherapy had a trend of reduced regional failure, but this trend did not reach statistical significance. (*p* = 0.32) [7]. As these studies were limited by small sample sizes or insufficient data about factors that could affect survival, this motivated us to use our national TCR database, which has both comprehensive clinicopathological information and enough patient numbers to assess this important issue in oral cancer management.

Our results showed that adjuvant radiotherapy is associated with significantly better 5-year OS (aHR, 0.72; 95% CI, 0.54–0.97; *p* = 0.0288) and DFS (aHR, 0.64; 95% CI, 0.48–0.84; *p* = 0.0016), after adjustment for confounders. Similarly to our findings, Qian et al. reported OS and DFS from single-center data and found that adjuvant radiotherapy was associated with significantly better OS (*p* = 0.04) and disease-free survival (DFS) (*p* = 0.04) in 221 patients with pT1-2N1 head and neck cancer [20]. Using the US Surveillance, Epidemiology, and End Results database, Shrime et al. and Torrecillas et al. both focused on patients with T1-2N1 oral cavity cancer, showing similar results [16,21]. Shrime et al. found that surgery with adjuvant radiotherapy significantly improved 5-year OS when compared with surgery alone (54.2% vs. 41.4%, respectively, *p* < 0.001). Torrecillas et al. also reported that adjuvant radiotherapy was associated with significantly better 5-year OS and 5-year DFS in all pT1-2 patients. Analyzing the different National Cancer Databases (NCDBs), Chen et al. and Suresh et al. likewise concluded that pT1-2N1 patients had better OS if they received adjuvant radiotherapy [22,23]. Recently, in another NCDB analysis, Xiang et al. observed that compared to postoperative observation, adjuvant radiotherapy was associated with improved survival (*p* = 0.03) [24]. In addition, among patients treated with adjuvant radiotherapy, the overall survival of pN1 was equivalent to pN0 and superior to pN2. It is worth mentioning that these studies investigating the results of the NCDB do not provide DSS. Ivaldi et al. conducted a meta-analysis which included 15 articles published in the last 10 years on early oral cavity cancer (pT1-2, N0-1) and summarized that those with a single positive neck node without extra-nodal extension or other adverse features, although rare, could benefit from adjuvant radiotherapy [18]. However, the limitation of the current meta-analysis is the lack of high-quality data to clearly define the role of adjuvant radiotherapy in such a clinical scenario.

Our results indicated that adjuvant radiotherapy could improve survival for patients with pT1-2N0 oral cancer, especially those with pT2 disease. Shrime et al. reported a survival advantage for 5-year OS and statistically significant DFS in those with pT2 classification disease (*p* < 0.001; *p* = 0.003), but not pT1 classification (*p* = 0.25; *p* = 0.35) [16]. Chen et al. also found that adjuvant radiotherapy was associated with better OS in patients with pT2 disease (HR, 0.64, 95% CI: 0.43–0.96), but not those with pT1 disease (HR, 0.80, 95% CI: 0.60–1.07) [22]. However, Torrecillas et al. reported that adjuvant radiotherapy was related to better 5-year OS and DFS in those with both pT1 (*p* = 0.007; *p* 0.001) and pT2 disease (*p* = 0.009; *p* = 0.004) [21]. These differences in outcomes may be due to the different populations studied or variety in the patient selection criteria. Moreover, these studies did not consider the quality of neck dissection, which may have led to the subsequent underestimation of disease severity [13]. For this reason, our observed estimated effect of adjuvant radiotherapy may be more accurate than that reported in the survival analysis of other studies.

In addition to local tumor extent as shown in Table 4, adjuvant radiotherapy was significantly associated with improved 5-year OS in females (aHR, 0.31 95% CI, 0.12–0.84; *p* = 0.0206), those aged ≥65 years (aHR, 0.29; 95% CI, 0.14–0.62; *p* < 0.012) and those with non-tongue disease (aHR, 0.62; 95% CI, 0.41–0.93; *p* = 0.0195); 5-year DFS in non-tongue disease (aHR, 0.61; 95% CI, 0.41–0.91; *p* = 0.0158). The possible reasons for this association are as follows. First, because of the high frequency of comorbidities in elderly patients, poor compliance to the use of adjuvant radiotherapy could cause worse overall survival [12,25]. Second, due to the relatively small number of female patients, the effect of adjuvant radiotherapy needs to be addressed in future investigations. In the literature, oral cancer originated from the tongue is more aggressive than other subsites and has the highest risk of nodal metastasis, which can range from 15 to 75%. Thus, an extension of lymph node therapy had been recommended [26]. However, our database cannot clearly define whether a primary tumor crossed the midline or not or whether the radiation filed to the ipsilateral or bilateral neck, which may underestimate the efficacy of adjuvant radiation for tongue cancer patients.

In the current study, among the 666 patients with risks (35 patients without recurrence data), 33 patients had tumor local recurrence, 43 patients had neck recurrences, 17 patients had local and regional recurrence and 15 patients had distant metastasis after initial surgery. Adjuvant radiotherapy provided higher control in neck recurrence (aHR, 0.30; 95% CI, 0.18–0.51; *p* < 0.001). Similarly, Shim et al. reported that there was no local failure in 86 T1-2N0-1 oral tongue cancer patients after adjuvant radiotherapy [16]. BG Weiss and colleagues also demonstrated that among 65 head and neck cancer patients with pT1-2N1 disease, adjuvant radiotherapy was associated with significantly better 5-year disease-specific survival, recurrence-free survival and higher local control rate (all, *p* < 0.05) [27]. The recent literature had reported that adequate neck dissection (LN ≥ 18) could improve neck control and increase survival; thus, adjuvant radiotherapy should not be administered for patients receiving high-quality neck dissection [5,13]. On the contrary, Suresh et al. reported that both adequate and inadequate dissections benefitted similarly from adjuvant radiotherapy in 1909 pT1-2N0-1 oral cancer patients. Interestingly, a recent retrospective analysis by Xiang et al. also found that adjuvant radiotherapy was beneficial in all LN removed subgroups (<10; 10–19; 20–29; ≥30) [24]. Consistent with our results, all this evidence elucidated that the role of adjuvant radiotherapy was not only to local recurrences but also to regional recurrences, even after adequate neck dissection.

There are several limitations to our study. As previously mentioned, although we excluded most of the risk factors related to the use of adjuvant radiotherapy, the lack of information on factors such as the depth of tumor invasion, whether it crossed midline or not, perineural invasion, and radiation field to ipsilateral or bilateral neck may have potentially confounded our analysis. Second, although HPV infection has recently been implicated in the pathogenesis and plays an important role in the survival of head and neck cancer patients, HPV status was not recorded in our national cancer registry database. A previous study had demonstrated that the prevalence of HPV infection in cases of oral cancer varies widely [28]. In Taiwan, oral cancer is often related to tobacco, alcohol, and betal nut chewing and is less associated with HPV [29,30]. Considering the effect of HPV infection, more retrospective studies from other countries are necessary to confirm our results. Despite these limitations, this study offers important and practical insight to help to guide adjuvant radiotherapy in early oral cancer.

## 5. Conclusions

By utilizing this national cancer registry database, adjuvant radiotherapy conferred an improved neck control and survival advantage in early oral cancer patients with single nodal status. These results suggest that the routine use of adjuvant radiotherapy should be considered, especially for those with pT2 and non-tongue disease.

## Figures and Tables

**Table 1:** Clinicopathological characteristics of study patients, *n* = 701.

Variation	RT		*p* Value *
	No (*n* = 196)	Yes (*n* = 505)	
	*n* (%)	*n* (%)	
Gender			
Female	20 (10.20)	53 (10.50)	0.9099
Male	176 (89.80)	452 (89.50)	
Age, years			
<65	155 (79.08)	448 (88.71)	0.0010
≥65	41 (20.92)	57 (11.29)	
AJCC pT			
T1	76 (38.78)	144 (28.51)	0.0086
T2	120 (61.22)	361 (71.49)	
Grade			
Well/moderately	179 (91.33)	425 (84.16)	0.0136
Poorly/undifferentiated	17 (8.67)	80 (15.84)	
Site			
Tongue	85 (43.37)	250 (49.50)	0.1443
Other	111 (56.63)	255 (50.50)	
CCI group			
0	133 (67.86)	387 (76.63)	0.0383
1	37 (18.88)	77 (15.25)	
≥2	26 (13.27)	41 (8.12)	
Hospital level			
Medical center	150 (76.53)	359 (71.09)	0.1471
Non-medical center	46 (23.47)	146 (28.91)	
Mortality	76 (38.78)	157 (31.09)	0.0525

* *p*-value was calculated from Pearson’s chi-square. Abbreviations: RT, radiotherapy; CCI, Charlson Comorbidity Index.

**Table 2:** Radiation treatment parameters.

Variation	*N*	%
RT dose, missing (*n* = 9)		
≤50 Gy	14	2.82
50–60 Gy	154	31.05
60–70 Gy	319	64.31
>70 Gy	9	1.81
RT techniques, missing (*n* = 11)		
3D	28	5.67
IMRT	422	85.43
VMAT	44	8.91
RT fields, missing (*n* = 9)		
Primary only	3	0.60
Neck only	3	0.60
Primary + neck	490	98.80

Abbreviations: RT, radiotherapy; IMRT, intensity modulated radiation therapy; VMAT, volumetric modulated arc therapy.

**Table 3:** Crude and multivariate hazard ratio in 5-year overall survival (OS) and disease-free survival (DFS), *n* = 701.

Variation	5-Year DFS				5-Year DFS			
	Crude HR (95% C.I)	*p* Value	Adjusted HR (95% C.I)	*p* Value	Crude HR (95% C.I)	*p* Value	Adjusted HR (95% C.I)	*p* Value
All								
RT	0.76 (0.57–1.01)	0.0596	0.72 (0.54–0.97)	0.0288	0.66 (0.50–0.87)	0.0030	0.64 (0.48–0.84)	0.0016
Age, ≥65	1.30 (0.90–1.88)	0.1570	1.25 (0.85–1.84)	0.2591	1.35 (0.95–1.91)	0.0986	1.30 (0.90–1.87)	0.1681
Male	1.17 (0.73–1.88)	0.5064	1.27 (0.78–2.08)	0.3447	1.27 (0.80–2.04)	0.3131	1.38 (0.85–2.25)	0.1960
pT2	1.81 (1.31–2.51)	0.0004	1.79 (1.29–2.50)	0.0006	1.61 (1.18–2.19)	0.0026	1.63 (1.19–2.23)	0.0024
Grade (Well/moderately)	0.62 (0.44–0.87)	0.0055	0.65 (0.46–0.91)	0.0135	0.67 (0.48–0.93)	0.0186	0.69 (0.49–0.98)	0.0369
CCI group								
0	Ref.		Ref.		Ref.		Ref.	
1	1.16 (0.81–1.67)	0.4181	1.14 (0.78–1.66)	0.4937	1.05 (0.73–1.51)	0.7789	1.01 (0.70–1.46)	0.9531
≥2	1.88 (1.28–2.77)	0.0014	1.75 (1.18–2.60)	0.0056	1.71 (1.16–2.51)	0.0064	1.58 (1.06–2.34)	0.0233
Hospital level, medical center	1.08 (0.79–1.46)	0.6347	1.06 (0.78–1.44)	0.7337	1.10 (0.81–1.47)	0.5464	1.06 (0.78–1.43)	0.7105
Site								
Tongue	1.04 (0.80–1.36)	0.7668	1.07 (0.81–1.40)	0.6423	1.10 (0.85–1.43)	0.4830	1.14 (0.88–1.49)	0.3244
Other	Ref.		Ref.		Ref.		Ref.	

Abbreviations: RT, radiotherapy; CCI, Charlson Comorbidity Index.

**Table 4:** Crude and multivariate hazard ratio in 5-year overall survival (OS) and disease-free survival (DFS) in different stratum groups, *n* = 701.

RT vs. Non-RT	5-Year OS				5-Year DFS			
	Crude HR (95% C.I)	*p* Value	Adjusted HR (95% C.I)	*p* Value	Crude HR (95% C.I)	*p* Value	Adjusted HR (95% C.I)	*p* Value
Gender								
Male	0.83 (0.62–1.13)	0.2419	0.80 (0.59–1.09)	0.1588	0.73 (0.55–0.98)	0.0349	0.71 (0.52–0.95)	0.0221
Female	0.32 (0.13–0.78)	0.0128	0.31 (0.12–0.84)	0.0206	0.25 (0.10–0.62)	0.0028	0.23 (0.09–0.61)	0.0033
Age, years								
<65	0.93 (0.67–1.30)	0.6734	0.88 (0.63–1.23)	0.4597	0.74 (0.54–1.01)	0.0560	0.70 (0.51–0.96)	0.0257
≥65	0.35 (0.17–0.70)	0.0028	0.29 (0.14–0.62)	0.0012	0.45 (0.23–0.86)	0.0160	0.42 (0.21–0.84)	0.0145
AJCC pT								
T1	1.00 (0.55–1.84)	0.9902	0.97 (0.51–1.83)	0.9230	0.79 (0.45–1.39)	0.4193	0.80 (0.44–1.44)	0.4509
T2	0.63 (0.45–0.87)	0.0053	0.65 (0.46–0.90)	0.0097	0.57 (0.41–0.77)	0.0004	0.58 (0.42–0.80)	0.0009
Site								
Tongue	0.86 (0.56–1.33)	0.5097	0.95 (0.61–1.49)	0.8288	0.65 (0.44–0.97)	0.0337	0.72 (0.48–1.09)	0.1194
Other	0.68 (0.46–1.00)	0.0491	0.62 (0.41–0.93)	0.0195	0.66 (0.45–0.97)	0.0328	0.61 (0.41–0.91)	0.0158

Abbreviations: RT, radiotherapy.

**Table 5:** Crude and multivariate hazard ratio in 5-year neck recurrence in different stratum groups, *n* = 666 (missing = 35).

Variation	Crude HR (95% C.I)	*p* Value	Adjusted HR (95% C.I)	*p* Value
All				
RT	0.29 (0.18–0.49)	<0.0001	0.30 (0.18–0.51)	<0.0001
Age, ≥65	1.89 (1.03–3.50)	0.0415	1.63 (0.86–3.10)	0.1333
Male	1.58 (0.57–4.35)	0.3788	1.86 (0.66–5.22)	0.2371
pT2	0.97 (0.56–1.65)	0.8968	1.10 (0.65–1.90)	0.7291
Grade (Well/moderately)	0.80 (0.41–1.59)	0.5282	0.88 (0.44–1.77)	0.7262
CCI group				
0	Ref.		Ref.	
1	1.25 (0.64–2.43)	0.5159	1.08 (0.55–2.13)	0.8297
≥2	1.59 (0.75–3.40)	0.2281	1.40 (0.64–3.04)	0.3962
Hospital level, medical center	1.48 (0.78–2.78)	0.2283	1.40 (0.74–2.66)	0.3010
Site				
Tongue	1.89 (1.12–3.19)	0.0176	2.10 (1.23–3.58)	0.0066
Other	Ref.		Ref.	

Abbreviations: RT, radiotherapy; CCI, Charlson Comorbidity Index.

## Data Availability

The datasets used during the present study are available from the corresponding author upon reasonable request.

## Supplementary Materials

The following are available online at https://www.mdpi.com/article/10.3390/cancers13153742/s1, Table S1: Number of patients by tumor subsite and radiation treatment.

## Abbreviations

TCR	Taiwan Cancer Registry
NHI	National Health Insurance
CCI	Charlson Comorbidity Index
TNM	tumor-node-metastasis
LN	lymph node
OS	overall survival
DFS	disease-free survival
HR	hazard ratio
CI	Confidence Interval
aHR	adjusted hazard ratio
NCDB	National Cancer Database
