# Supplementary material for: Adjuvant Radiotherapy Significantly Increases Neck Control and Survival in Early Oral Cancer Patients with Solitary Nodal Involvement: A National Cancer Registry Database Analysis
Source: Cancers (Basel). 2021 Jul 26;13(15):3742. doi: 10.3390/cancers13153742 (PMC8345217; doi:10.3390/cancers13153742)
Supplement: Supplementary file 1 [file cancers-13-03742-s001.zip › cancers-1254997-supplementary.pdf]

cancers

MDPI

Supplementary Materials

Adjuvant Radiotherapy Significantly Increases Neck Control and Survival in Early Oral Cancer Patients with Solitary Nodal Involvement: A National Cancer Registry Database Analysis

Chia-Jen Tsai, Yu-Hsuan Kuo, Hung-Chang Wu, Chung-Han Ho, Yi-Chen Chen and Ching-Chieh Yang

Table S1. Number of patients by tumor subsite and radiation treatment

**Variation**	**Total**	**RT**	
		**No**	**Yes**
	***n* = 701**	***n* = 196**	***n* = 505**
Total			
Tongue (C02)	335 (47.79)	85 (43.37)	250 (49.50)
Gum (C03)	20 (2.85)	6 (3.06)	14 (2.77)
Floor of mouth (C04)	28 (3.99)	9 (4.59)	19 (3.76)
Palate (C05)	16 (2.28)	5 (2.55)	11 (2.18)
Other parts of the mouth (C06)	302 (43.08)	91 (46.43)	211 (41.78)

Lip (excluding skin of lip) (C00), *n* = 0. Abbreviation: RT, radiotherapy.
